# Supplementary material for: Netcombin: An algorithm for constructing optimal phylogenetic network from rooted triplets
Source: PLoS One. 2020 Sep 18;15(9):e0227842. doi: 10.1371/journal.pone.0227842 (PMC7500971; doi:10.1371/journal.pone.0227842)
Supplement: S4 File — (ZIP) [file pone.0227842.s004.zip › SequenceData-Triplet/sequenceData-10-20-30-0-Triplet/length 500, numseq 40, rate 10^-7/2 seq.pdf]

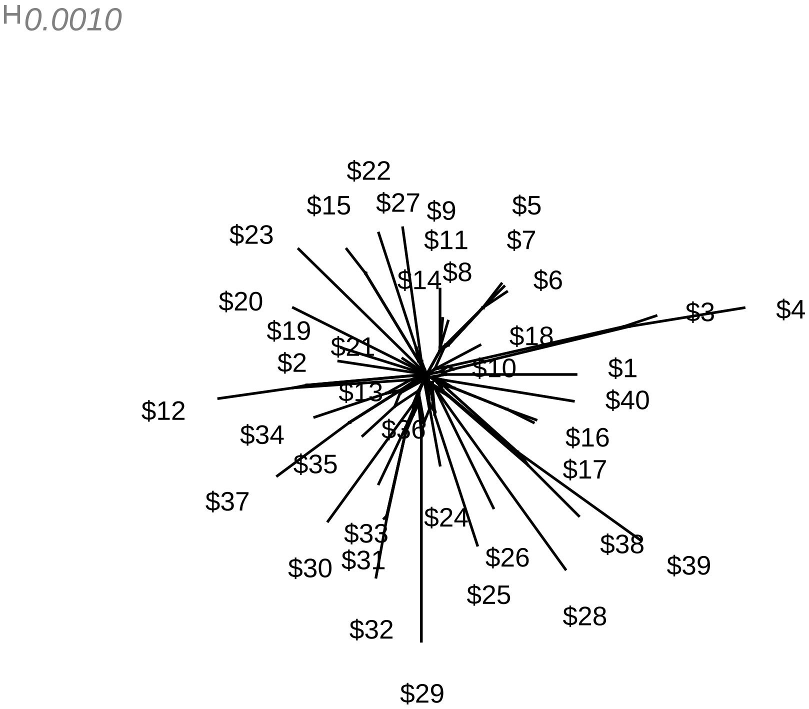
0.0010
$22
$15
$27
$9
$5
$23
$11
$7
$14
$8
$6
$20
$3
$4
$19
$18
$21
$2
$10
$1
$13
$40
$12
$34
$36
$16
$35
$17
$37
$24
$33
$38
$31
$26
$39
$30
$25
$28
$32
$29
